# Secured regions of interest (SROIs) in single-pixel imaging

**DOI:** 10.1038/s41598-019-49282-y

**Published:** 2019-09-04

**Authors:** Zhiyuan Ye, Bo Su, Panghe Qiu, Wenxiang Gao

**Affiliations:** 1 Key Laboratory of Terahertz Optoelectronics, Ministry of Education, Beijing, 100048 China; 2 Beijing Key Laboratory for Terahertz Spectroscopy and Imaging, Beijing, 100048 China; 3 Beijing Advanced Innovation Centre for Imaging Theory and Technology, Beijing, 100048 China; 4 Department of Physics, Capital Normal University, Beijing, 100048 China

**Keywords:** Optics and photonics, Physics

## Abstract

Single-pixel imaging, which is also known as computational ghost imaging, can reconstruct an entire image using one non-spatially resolved detector. However, it often requires a large amount of sampling, severely limiting its application. In this paper, we discuss the implementation of secured regions of interest (SROIs) in single-pixel imaging and illustrate its application using two experiments. Under a limited number of sampling times, we improved the resolution and recovered spectral information of interest in the ROI. Meanwhile, this scheme has high information security with high encryption and has great potential for single-pixel video and compressive multi-spectral single-pixel imaging.

## Introduction

Single-pixel imaging^1^, which is also referred to as computational ghost imaging^2^, is a type of indirect imaging technique that uses one non-spatially resolved detector to reconstruct an entire image. In recent years, single-pixel imaging has succeeded in multi-spectral imaging^3–5^, three-dimensional imaging^6,7^, imaging at non-visible wavelengths^8–12^, optical encryption^13,14^, and imaging under conditions of low light^15^ or in environments with high noise^16–19^.

Generally, single-pixel imaging requires a sequence of structured light patterns to be projected onto an object, and the light intensity is measured by a single-pixel detector. Initially, these light patterns are generated by rotating frosted glass; then, a spatial light modulator and digital micromirror devices (DMDs) are used to artificially generate illumination patterns. Furthermore, the imaging quality and efficiency is closely related to the illumination pattern model used. Pseudo-inverse ghost imaging^20^, sinusoidal ghost imaging^21^, and Hadamard single-pixel imaging^22–24^ have been proposed to improve the imaging quality by increasing the orthogonality of the model. In addition, the recently reported Fourier single-pixel imaging^19^ uses sinusoidal intensity patterns, where the light intensity represents the coefficient of the Fourier transform of the image.

For all of the above strategies, a large number of illumination patterns cannot be avoided according to Nyquist’s sampling law, even if some strategies are highly compressive or underdamped and dictate that a decrease in the number of samples is at the expense of either the signal-to-noise ratio (SNR) or resolution^25^. The recently proposed single-pixel video^26,27^ method depends heavily on the modulation speed of the DMD and high compression of data. Hence, it is critical to select suitable schemes^28,29^ to compress big data or flexibly coordinate the relationship among the SNR, resolution, and imaging speed.

Regions of interest^30–32^ (ROIs) are extremely useful in image and video coding. Their use optimizes the coding performance, decreases the processing time and bandwidth usage, and increases the accuracy of specific areas. In 2017, David B. Phillips *et al*. adopted a novel strategy to inspire the animal’s foveated vision systems to implement dynamic ROIs in single-pixel imaging^29^. Different from ref.^29^, a new coding scheme is adopted to introduce secured Regions of interest (SROIs) in single-pixel imaging. This scheme has high encryption performance while implementing dynamic ROIs and can be extended to full-color or multi-spectral single-pixel imaging.

In this paper, we discuss in detail the implementation of secured regions of interest (SROIs) in single-pixel imaging. In our experiments, we used two different setups to illustrate the applications of SROIs. Experiment A included a DMD to generate binary illumination patterns based on the Hadamard matrix, and Experiment B included a digital light projector to play composite color illumination patterns based on the Hadamard matrix. In particular, the Hadamard matrix was linearly combined into illumination patterns of unevenly distributed sizes and colors, which improved the resolution and spectral information of the imaging area of interest. Meanwhile, this linear mapping method has a high randomness. We wrote this randomness into a multi-colored cipher pattern, and only the user with the cipher pattern can decrypt the correct image. All kinds of illumination patterns can be displayed onto objects, and a single-pixel detector records the light intensities. We can then use a simple and fast algorithm to recover the object. A large number of experiments using this scheme, such as single-pixel imaging of biological tissues, real-time tracking imaging of moving targets, and multi-spectral image fusion are proposed, which all confirm the feasibility of this scheme.

## Methods

### Single-pixel imaging based on the Hadamard matrix

Single-pixel imaging requires a series of structured light patterns to illuminate a target and simultaneously collect the relative light intensity with an amplified photodetector (APD). We can use an *N*-dimensional column vector, **o**, to represent the target for imaging, where *N* represents the number of pixels of the recovered image. We use an *n* × *n* matrix, **h**, to represent the Hadamard basis and use the equation ${{\bf{h}}}_{ij}={\bf{h}}(:,i){\bf{h}}(j,:)$ to generate a series of mask patterns, where ${\bf{h}}(:,i)$ and ${\bf{h}}(j,:)$ $(1\le i\le n,1\le j\le n,N={n}^{2})$ represent the *i-th* column and the *j-th* row of matrix **h**, respectively. We reshape all mask patterns, ${{\bf{h}}}_{11},{{\bf{h}}}_{12},\ldots ,{{\bf{h}}}_{nn}$, into row vectors, ${{\bf{H}}}_{1},{{\bf{H}}}_{2},\mathrm{...},{{\bf{H}}}_{N}$, and form *N* such row vectors into a measurement matrix **H**. Because the Hadamard basis **h** is an orthogonal matrix, **H** is also an orthogonal matrix, i.e., ${\bf{H}}{{\bf{H}}}^{T}={{\bf{H}}}^{T}{\bf{H}}={\bf{I}}$, where **H**^*T*^ represents the transpose of matrix **H**, and **I** represents the scalar matrix. This physical process can be described by the following equation:

1 $${\bf{b}}={\bf{H}}{\bf{o}},$$

where **b** is an *N*-dimensional column vector that represents the relative intensity values measured by the APD. The elements in a Hadamard matrix take values of +1 or −1. The DMD consists of millions of reversible micromirrors and can play illumination patterns that transmit (micromirror “on”) or block (micromirror “off”) intensity regions. A micromirror that is “on” represents a value of +1, and a micromirror that is “off” represents a value of 0, which means that −1 in **H** is represented by 0. As a result, we can use equation ${\bf{B}}=2{\bf{b}}-{b}_{0}$ as compensation, where *b*_0_ represents the relative light intensity with all micromirrors on. Thus, we can use the following equation to reconstruct the image:

2 $$\hat{{\bf{o}}}={\bf{H}}({\bf{B}}+{\bf{c}}),$$

where $\hat{{\bf{o}}}$ represents the estimated solution of **o**, and **c** represents the noise that is inevitably brought about via the actual measurement of **b**. Note that the number of the light patterns is equal to the pixels of the image to be reconstructed. For a known measurement system, equation $SNR=20{\log }_{10}\langle {\bf{b}}\rangle /\langle {\bf{c}}\rangle$ describes the SNR of the system, where $\langle \rangle$ represents the average magnitude of all elements in the corresponding vector and the voltage amplitude of the measurement system when there is no signal input that can be seen as the noise, $\langle {\bf{c}}\rangle$.

### The implementation of SROIs in Experiment A

**Figure 1:**
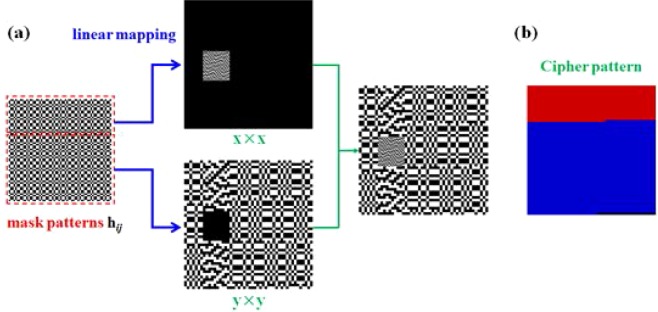
(**a**) Schematic diagram of generating a composite light pattern (64 × 64) in Experiment A and (**b**) cipher pattern corresponding to the linear mapping method.

As shown in Fig. 1(a), the original mask patterns were linearly mapped to new light patterns consisting of two different unit sizes, *x* × *x* and *y* × *y*. Thus, the size of some of the elements in the original illumination patterns decreased and constituted the ROI of the composite illumination patterns, while the size of other elements increased and constituted the other part of the composite illumination patterns. However, the unevenness of the size resulted in linear distortion of the intensity during the physical imaging process. We can multiply the imaging results of ROI by (*y*/*x*)^2^ to simply eliminate the linear distortion. Meanwhile, this linear mapping method is highly random, and we can use this artificial randomness as the secret key to providing the correct image. The position of the red region in Fig. 1(b) corresponds to the index position of the elements in the original illumination patterns in the ROI region, the blue region corresponds to the other regions, and the black position is an invalid region. The decryption process performs inverse linear mapping according to the cipher pattern after obtaining the imaging result using the above equation.

### The implementation of adaptive SROIs in Experiment B

**Figure 2:**
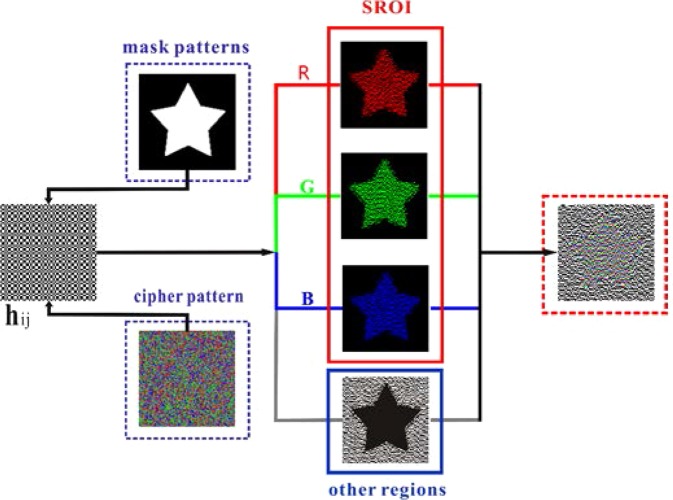
Schematic diagram of generating a composite color illumination pattern (64 × 64) in Experiment B.

In Experiment B, we used a digital light projector to play multi-colored illumination patterns onto the object. As shown in Fig. 2, we used an arbitrary mask template as the shape of the SROI and used a multi-color cipher pattern to record the linear mapping method. This cipher pattern is similar to the one discussed above. Red, green, and blue correspond to the three RGB channels of the SROI, respectively, and gray corresponds to other regions, whereas black corresponds to redundant elements. The mask template and the cipher pattern are closely related to the generation of color composite illumination patterns based on the Hadamard matrix. Using this method, we obtained the multi-spectral resolution of the region of interest while other regions were disregarded.

### Experimental setups

**Figure 3:**
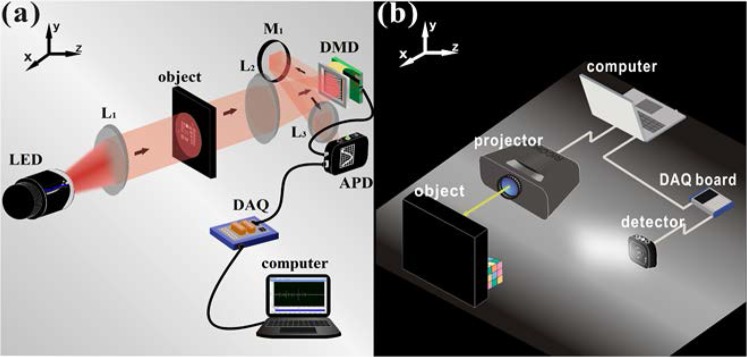
Schematic diagrams of the two experimental setups.

The setups for Experiment A and Experiment B are shown in Fig. 3. Both experimental setups consisted of an illumination system and an acquisition measurement system, which shared the same acquisition measurement system, including an APD (Thorlabs PDA100A2), a shielded connector block (National Instruments BNC-2110), and a data acquisition card (National Instruments PCI-6220). In Experiment A (see Fig. 3(a)), the light emitted by the LED (Thorlabs M850L3 850 nm) was collimated by lens L_1_ and then illuminated on the object (resolution board: USAF 1951). Then, the beam was illuminated on the DMD (Texas Instruments DLPA008A) through lens L_2_ in which the position of the target and the position of the DMD satisfied the imaging relationship. The DMD was controlled by the computer and stored a series of binary mask patterns in advance. The beam was modulated by the DMD and collected by lens L_3_ on the target surface of the APD, which converted the optical signals into electrical signals and amplified them. When the DMD played speckle patterns at a certain frame rate, it sent synchronous trigger signals to the shielded connector block, which synchronized the voltage signal transmitted by the APD. Finally, the collected voltage signal was converted into a digital signal by the DAQ card and transmitted to the computer for processing. In Experiment B (see Fig. 3(b)), the digital light projector (RICOH PJ S2440) projected a mixed color image onto the object, whose diffuse light was captured by the same acquisition measurement system.

## Results

### Imaging results from Experiment A

In Experiment A, we activated the central area (768 × 768 micromirrors) of the DMD as the modulation area. The playback rate of the DMD was set to 2.5 kHz, and the sampling rate of the DAQ was set at 200 kS/s. The SNR of this system can reach 54 dB (the average voltage amplitude of the signal was approximately 5 V, and the average amplitude of the background noise was approximately 10 mV).

**Figure 4:**
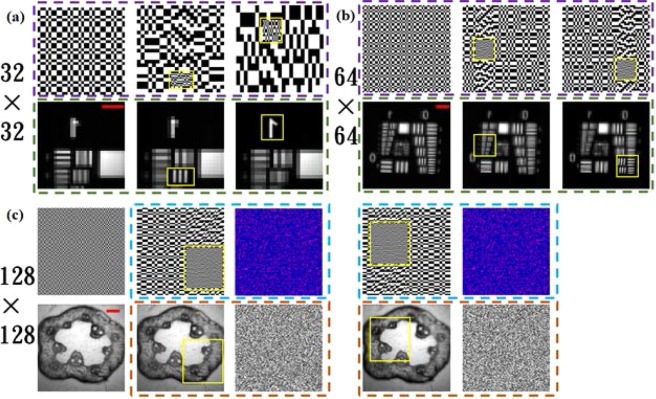
Imaging results for Experiment A. The area selected by the yellow box is where the ROI lies. The scale bar in red = 1 mm. (**a**) The number of light patterns based on the Hadamard matrix was 1024, and three kinds of light patterns and their corresponding imaging results are shown. (**b**) The number of light patterns based on the Hadamard matrix was 4096, and three kinds of light patterns and their corresponding imaging results are shown. (**c**) The target was crosscut pumpkin stems, and the number of the light patterns was limited to 16384. The image in the blue dotted frame is followed by a linearly mapped illumination pattern and a cipher pattern, and the corresponding red dotted frame image was the result of decryption using the correct cipher pattern and the result obtained using the wrong cipher pattern.

As shown in Fig. 4(a), the number of light patterns based on the Hadamard matrix was 32 × 32 and three kinds of light patterns and their corresponding imaging results are shown. The first column recovers an image of the object with the resolution of 32 × 32 using the original illumination patterns. In the illumination patterns of the second and third columns, the cell of the ROI consists of 8 × 8 micromirrors, while the basic unit of the other regions consists of 32 × 32 micromirrors; thus, the resolution of the ROI is four times more than that of the other areas. As shown in Fig. 4(b), similar to the results shown in Fig. 4(a), the number of light patterns is 64 × 64, and the first column recovers an image of the object with the resolution of 64 × 64 using the original illumination patterns. The cell of the ROI consists of 4 × 4 micromirrors, whereas the cells of the other regions consists of 16 × 16 micromirrors. From the results of the image, the resolution of the ROI was considerably improved compared to the original image for a limited sampling rate.

To further illustrate the practical application of this approach, we used this scheme to image biological tissue. In real-time biological tissue imaging, there are often stringent requirements for resolution and imaging quality, which may pose serious challenges for single-pixel imaging. Under the premise of having a low-resolution preview of the biological tissue to be imaged in advance, we used this scheme to achieve high-resolution encrypted imaging of the ROI. As shown in Fig. 4(c), we selected crosscut pumpkin stems as the imaging target. The number of light patterns was limited to 16384, and the first column recovered an image of the object with the resolution of 128 × 128 using the original light patterns in which we combined 6 × 6 micromirrors into one basic unit. In the new light patterns after being linearly mapped, the cell of the ROI consisted of 3 × 3 micromirrors, while the cell of the other regions consisted of 12 × 12 micromirrors. Thus, the resolution of the ROI was four times higher than that of other areas. Meanwhile, the linear mapping method was recorded as a cipher pattern (see Methods). If the correct cipher pattern is unknown, this will result in a completely incorrect image.

In a single-pixel video^26,27^, a large number of samples are equally inevitable, and it is the modulation speed that determines the frame rate and video resolution. The fastest modulation speed of the DMD used in Experiment A was 2.5 kHz, which theoretically could achieve a video frame rate of one frame per second for a resolution of 50 × 50. For making the resolution of the local area exceed this limit, our proposed SROI scheme was found to be effective. In fact, existing high-performance DMDs are already capable of modulating at 22 kHz, or in one particular case, a group has developed an LED-based structured illumination with the modulation rate of 250 kHz^33^. Due to the limitations of inherent hardware (such as advanced DMD and high-speed response acquisition equipment), we selected the modulation rate of 2.5 kHz to conduct a proof-of-principle experiment.

**Figure 5:**
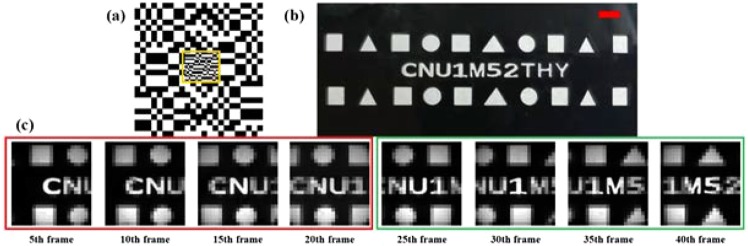
Experimental results of a single-pixel video with a static ROI. (**a**) Light patterns for the ROI (the yellow border selected area). (**b**) Object and scale bar in red = 4 mm. (**c**) Every fifth frame of the video (see Supplementary Video 1 for the complete video).

**Figure 6:**
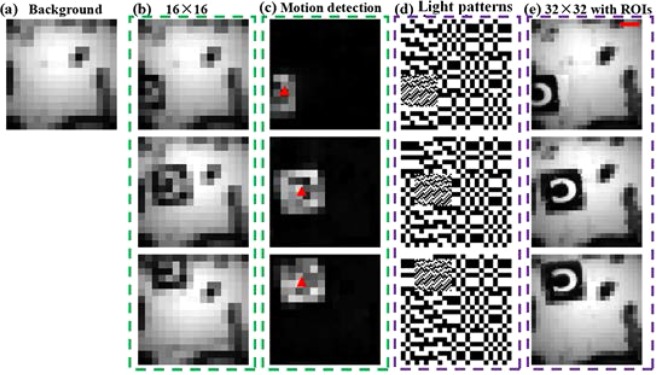
Experimental results for the single-pixel video with an intelligent and dynamic ROI. **(a**) The background item was collected in advance (at a resolution of 16 × 16). (**b**) The low-resolution single-pixel video (16 × 16) used to quickly detect moving targets. (**c**) Background difference between (**a**,**b**), where the small red triangle is the center position of the moving target estimated in (**c**) by a series of image processing operations. (**d**) Speckle pattern for generating the next frame based on the position and velocity of the moving target in (**c**). (**e**) The high-resolution single-pixel video (32 × 32) with ROIs, and the scale bar in red = 2 mm. (**b**–**d**) All include three sets of frames taken from the single-pixel video in Supplementary Video 2.

As shown in Fig. 5, we applied a static ROI to single-pixel video, and we set the position of the ROI to be in the center of the screen. Owing to hardware limitations, the frame rate was only two frames per second (fps); thus, we chose a slow-moving object as the imaging target (see Fig. 5(b)). Supplementary Video 1 shows the single-pixel video for a static ROI with a total duration of 20 s and a total of 40 frames. Figure 5(c) shows a group of pictures extracted from the video every five frames. As shown from the experimental results, we improved the resolution of the ROI to 96 × 96, which is three times better than the original resolution. To be more practical, we applied an intelligent dynamic ROI to the single-pixel video. In the following example, we used the same scheme to encode the illumination mode using background subtraction^34^ to achieve single-pixel video detection and tracking of moving targets to improve the resolution of the moving targets. As shown in Fig. 6 and Supplementary Video 2, we first collected the background item at a resolution of 16 × 16 so that we could achieve a frame rate of 8 fps to detect whether there were moving objects. When a moving target was detected, the center position of the target and the estimated speed were obtained by image processing, such as morphological processing, thereby generating a new pattern. We further obtained a single-pixel video with the ROIs. The area of the ROI was roughly the area in which the moving target was located; thus, this simple example demonstrates high-resolution tracking of a moving target under a limited number of sampling times. It also suggests that the algorithms and strategies that are applicable to an area array detector can also be applied to single-pixel video after it is adjusted and modified.

### Imaging results from Experiment B

In Experiment B, the total number of illumination patterns played by the digital light projector was 128 × 128, and the playback speed was 30 Hz. Thus, 16384 illumination patterns were made into a 30-frame video in avi format and played onto the object. The SNR of this system could reach 31.5 dB (the average voltage amplitude of the signal was approximately 4.5 V, and the average amplitude of the background noise was approximately 0.12 V).

**Figure 7:**
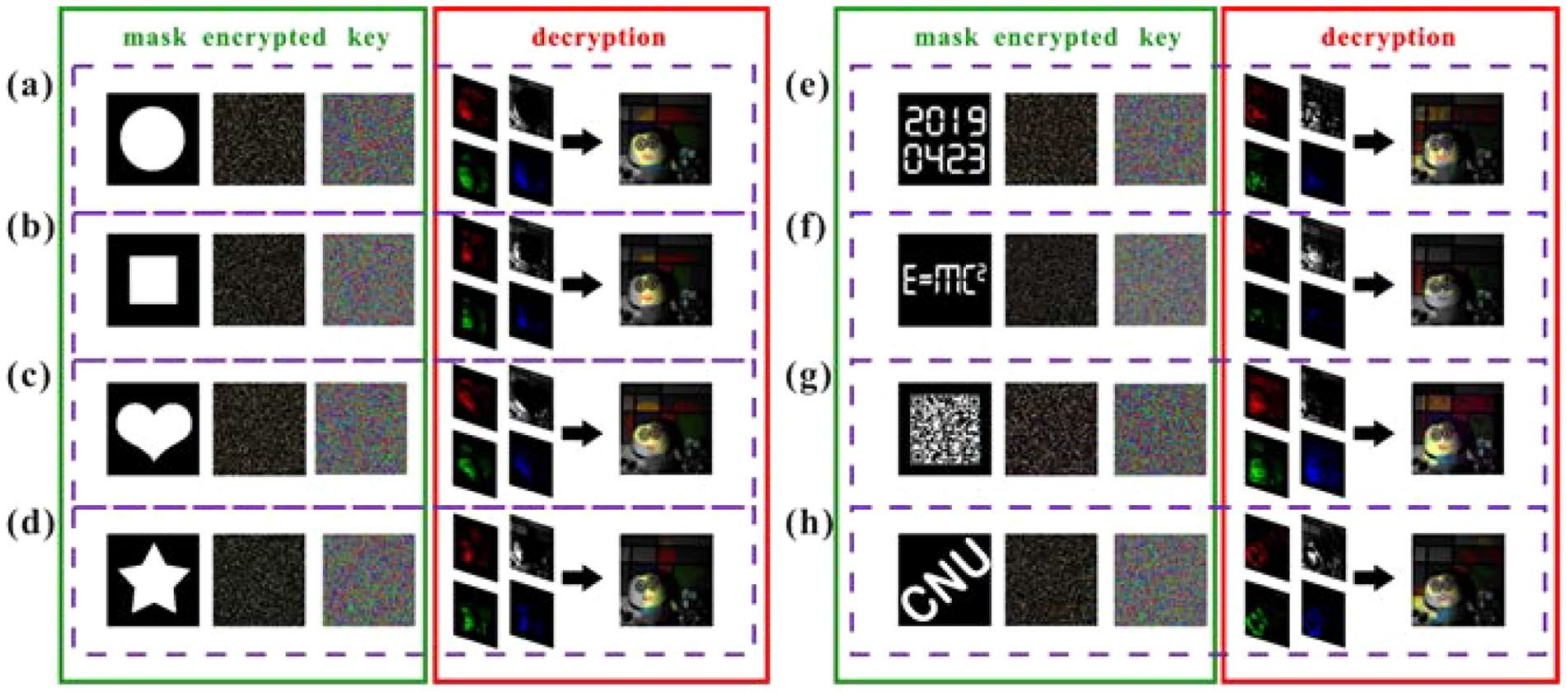
Imaging results for Experiment B. A total of eight sets of experimental results are presented. In the green solid line box are the mask templates, the encrypted imaging results, and the multi-color cipher patterns. The decryption is in the red solid line box. The three channels, which were linearly mapped into the SROI according to the cipher pattern and the gray-scale channel of other regions, finally produced the composite imaging results.

In Experiment A, we achieved an increase in resolution for the ROI with encryption. In this experiment, we recovered some spectral information about the ROI with encryption using single imaging. As shown in Fig. 7, we used a three-dimensional color scene as the imaging target and gave a total of eight sets of experimental results using different shapes of the SROI. As shown from the experimental results, we initially recovered the color information or some spectral information in the SROI, and the high randomness of the cipher pattern provided the high information security for the scheme. We also found that the shape of the mask template can be naturally loaded as a watermark pattern in the composite imaging results (see Fig. 7(e–h)). We admit that there is a color difference in the color information of the imaging result because the APD responds differently to the three main bands of the digital projector; however, this can be corrected by digital processing.

## Discussion

In single-pixel imaging, under-sampling results in a decrease in SNR or resolution, whereas full sampling increases the imaging time. In this paper, we proposed an alternative technology called SROI, which has been experimentally confirmed to improve the resolution of the ROI or make the ROI a certain spectral resolution. This technology has high scalability and high encryption, and the shape of the ROI can be adaptive and can be used as optical watermarking. Where high volume data acquisition is required, such as single-pixel video or single-pixel multi-spectral imaging, specific information about the ROI can be obtained at a high quality. The under-sampling mechanism of SROI is not discussed in this paper because it is essentially one of the under-sampling strategies used to balance the sample rate and the resolution with a high SNR. In theory, this scheme can be implemented at a much lower sampling number (that is, the sampling number is lower than the number of pixels), because the Hadamard transform is similar to the Fourier transform, and the natural object’s information is mainly concentrated in a small part of coefficients^23,35^. Of course, this technology also faces some challenges. For example, in Experiment A, although the DMD could achieve single-pixel imaging at a low video frame rate, it did not spatially resolve the spectrum. In Experiment B, the commercial digital projector was unable to achieve accurate spectral segmentation, and its playback frame rate was very slow, which resulted in long-term acquisition; thus, it may limit the imaging of a mixed spectrum of dynamic scenes.

In conclusion, this study proposes a method for specifying SROIs in single-pixel imaging. We used a high encryption coding method, which involves linear mapping of the Hadamard matrix, to redistribute the resolution and color information, improving the resolution of the ROI and restoring its color information or certain spectral information. Random linear mapping can be recorded as a cipher pattern and will considerably improve the information security of imaging. Moreover, we applied this technology to single-pixel video to realize a tracking image of a moving target using a static ROI and intelligent and dynamic ROI. This technology is an under-sampling strategy that can be used to balance the sample rate and resolution with a high SNR, and it has considerable potential for use in applications, such as secured single-pixel imaging of biological tissue, single-pixel video of moving targets, and secured single-pixel multi-spectral imaging of static scenes.

## Supplementary information

Video S1

Video S2
